# Gap Junctions or Hemichannel-Dependent and Independent Roles of Connexins in Fibrosis, Epithelial–Mesenchymal Transitions, and Wound Healing

**DOI:** 10.3390/biom13121796

**Published:** 2023-12-14

**Authors:** Yuting Li, Francisca M. Acosta, Jean X. Jiang

**Affiliations:** 1 Department of Biochemistry and Structural Biology, University of Texas Health Science Center at San Antonio, San Antonio, TX 78229, USA; liyuting95@163.com (Y.L.); acostafm@uthscsa.edu (F.M.A.); 2 Department of Pathology, Basic Medical School, Ningxia Medical University, Yinchuan 750004, China

**Keywords:** connexins, gap junctions, hemichannels, fibrosis, EMT, wound healing

## Abstract

Fibrosis initially appears as a normal response to damage, where activated fibroblasts produce large amounts of the extracellular matrix (ECM) during the wound healing process to assist in the repair of injured tissue. However, the excessive accumulation of the ECM, unresolved by remodeling mechanisms, leads to organ dysfunction. Connexins, a family of transmembrane channel proteins, are widely recognized for their major roles in fibrosis, the epithelial–mesenchymal transition (EMT), and wound healing. Efforts have been made in recent years to identify novel mediators and targets for this regulation. Connexins form gap junctions and hemichannels, mediating communications between neighboring cells and inside and outside of cells, respectively. Recent evidence suggests that connexins, beyond forming channels, possess channel-independent functions in fibrosis, the EMT, and wound healing. One crucial channel-independent function is their role as the primary functional component for cell adhesion. Other channel-independent functions of connexins involve their roles in mitochondria and exosomes. This review summarizes the latest advances in the channel-dependent and independent roles of connexins in fibrosis, the EMT, and wound healing, with a particular focus on eye diseases, emphasizing their potential as novel, promising therapeutic targets.

## 1. The Role of Connexins in Fibrosis, EMTs, and Wound Healing

### 1.1. Structure of Connexin

Connexins are transmembrane proteins. Approximately 20 types of connexin genes have been reported in humans and mice [1,2]. The corresponding genes are abbreviated as “GJ” (for gap junction), while the most commonly used protein nomenclature employs “Cx” (for connexin). Connexin proteins are often identified by the molecular mass of the predicted polypeptide in kilodaltons (e.g., connexin43, Cx43) [3]. Different connexin sequences have been used to recognize connexin subfamilies, with five connexin subfamilies defined (α, β, γ, δ, and ε in mice or GJA, GJB, GJC, GJD, and GJE in humans) [1], each of them containing distinct biophysical properties and expression patterns with various biological functions. All connexins have similar topological structural characteristics, consisting of four transmembrane domains: the amino (NT) and carboxyl termini (CTs) in the cytoplasm, a cytoplasmic loop (CL), and two extracellular loops (EL1 and EL2) [4,5]. The CTs are considered regulatory regions for multiple post-translational modifications and protein binding sites, exhibiting the greatest heterogeneity in amino acid sequences [6]. Every six connexin monomers form a hexagonal lattice of protein subunits, as seen under electron crystallography, called connexons or hemichannels (HCs) [7]. After trafficking to the membrane docks, two HCs from adjacent cells with a “head-to-head” pattern form an intercellular channel called a GJ [8]. These GJs and HCs allow the passage of small molecules (Mw < 1 kDa), including ions, essential metabolites, and second messengers such as Ca^2+^, IP3, nicotinamide adenine dinucleotide (NAD), prostaglandin E_2_ (PGE_2_), ATP, ADP, cAMP, and cGMP [9]. This type of intercellular communication is known to be essential for various physiological activities, including cell growth, proliferation, and differentiation. It also plays crucial roles in tissue homeostasis, tumorigenicity, fibrosis, and wound healing.

### 1.2. Fibrosis and EMTs

Fibrosis is characterized by an excess accumulation of fibrous connective tissue in an organ. It is defined by the pathological assembly of extracellular matrix (ECM) components, which, over time, leads to scar tissue formation and ultimately affects organ function [10]. The epithelial–mesenchymal transition (EMT) is characterized by a cell’s switch from an epithelial state to a mesenchymal one during physiological and pathological events [11,12]. During the EMT, a polarized epithelial cell, which generally interacts with the basement membrane via its basal surface, undergoes multiple biochemical changes, resulting in it acquiring a mesenchymal phenotype. The mesenchymal phenotype involves increased migratory capacity, invasiveness, elevated resistance to apoptosis, and a largely incremental ECM [13]. The EMT is classified into three subtypes: The Type-1 EMT, which is involved in embryogenesis and organ development, and the Type-3 EMT, which is implicated in cancer progression [14]. Here, we will focus on the Type-2 EMT, associated with wound healing, tissue regeneration, and organ fibrosis.

### 1.3. GJ and HC in Fibrosis, EMTs, and Wound Healing

During fibrogenesis, fibroblasts are activated by various stimuli and require a complex system of signals to be received and transmitted. Due to their ability to permit small molecules to diffuse from one cell to another, GJs play a critical role in regulating tissue homeostasis and various processes responsible for restoring the homeostatic balance following damage, including wound healing and tissue repair [15]. Furthermore, in recent years, HCs have been found to have functions different from GJs by mediating communication between the intracellular compartment and the extracellular environment [16]. The molecules that diffuse through HCs are similar to those involved in GJs, including ATP, NAD, glutamate, glutathione, and prostaglandins [17]. Meanwhile, HCs open in pathological and normal physiological conditions and play a major role in maintaining tissue homeostasis [18,19]. Additionally, some connexins have channel-independent functions that are often associated with their ability to bind and organize cytoskeletal and signaling elements [20]. Therefore, their dysfunction is also associated with wound healing, tissue regeneration, and organ fibrosis [8]. In this review, we will discuss both channel-dependent and channel-independent roles in various tissues in fibrosis, the EMT, and wound healing.

## 2. Channel-Dependent Functions of Cx in Fibrosis, EMTs, and Wound Healing

### 2.1. Connexin GJs and HCs in Eye Diseases

#### 2.1.1. Cornea

The eye comprises the cornea, lens, uvea, retina, and several other subcomponents, within which various connexins exist. GJ communication in the human corneal epithelium is mediated by Cx26, Cx30, Cx31.1, and Cx43 [21,22]. Specifically, Cx43 has been associated with inflammatory responses and wound healing in injured corneas [23,24]. Cx43 knockdown can accelerate wound healing in the corneal endothelium and promote re-epithelialization by suppressing stromal oedema and inflammatory responses [25,26]. The interaction between Cx43 and zonula occludens-1 (ZO-1), a scaffolding protein that plays a role in regulating GJ assembly, has been extensively studied [27,28,29,30]. The alpha-carboxy terminus 1 (αCT1) peptide, derived from the carboxyl terminus of Cx43, disrupts Cx43 interaction with ZO-1, leading to an enhancement of GJs [31]. Treatment with αCT1 may play a role in the early stages of migration during corneal healing, as well as in affecting EMT pathway genes [32,33]. Cx43 antisense oligodeoxynucleotides (AsODNs), which knockdown Cx43 expression, have been shown to have a definite therapeutic effect in a reproducible model of corneal wound healing [26]. Cx43 AsODN-treated corneas show improved corneal clarity, faster re-epithelialization, and reduced inflammation. Additionally, the reduction in Cx43 GJ function with AsODNs in human nonhealing ocular burn wounds could enhance the recovery of limbal reperfusion and reduce inflammation, holding therapeutic potential for the treatment of severe, often unresponsive corneal injuries. This is achieved by targeting the downregulation of Cx43 to enable vascular recovery, making Cx43 a possible key factor in the treatment of wounds [34]. Moore et al. also found a downregulation of Cx43 in corneal wound healing. Between the 24- and 72-h time points, as the wound is healing, Cx43 is upregulated as cell–cell adhesion and GJ formation recur. By day 21, there was a downregulation in Cx43 [32]. The critical point is the time for treatment. Early use increases GJs, which are closely related to inhibiting inflammation and reducing edema. But in later stages, reducing over-expressed Cx43 can promote repair, speed healing, and prevent excessive scarring. Gap27 is a mimicking sequence on the second external loop of the Cx43 HC [9,35]. Its use in the early stages of wound healing for superficial corneal epithelial wounds has been proposed for promoting corneal epithelial healing, including persistent corneal ulcers, limbal stem cell deficiency, and dry eye syndrome. However, prolonged and frequent treatment in stromal wounds could elicit an undesirable inflammatory response [36].

#### 2.1.2. Lens

The lens epithelial cells express Cx43 and Cx50, while fiber cells express Cx46 and Cx50 [37]. Past research primarily focuses on the function and regulation of GJs because these channels play essential roles in maintaining lens cell homeostasis, metabolic coupling, and preventing the accumulation of reactive oxidants. Connexin mutations are being identified and linked to numerous types of human congenital cataracts [38,39]. Abnormal activities of GJs and/or HCs would greatly compromise these crucial functions. Our group has previously reported that the G143R mutation increases the interaction between the intracellular loop domain and Cx46, which is associated with the reduction in GJs but an increase in HC function [40]. Additionally, Cx50 HCs activated by H_2_O_2_ mediate glutathione (GSH) transport and protect lens fiber cells from oxidative stress [41]. Mechanically activated Cx HCs and GSH transport can reduce H_2_O_2_- and UVB-induced intracellular reactive oxygen species (ROS), further mitigating cellular apoptosis and death [42]. Our recent study confirmed that protein kinase A (PKA) activation reduces cataracts induced by oxidative stress, increases GJs/HCs in Cx50, Cx46, or Cx50 and Cx46 co-expressing cells, and decreases ROS levels and cell death [43]. 

We proposed a new molecular mechanism of HCs in lens epithelial cells that protects the lens against oxidative stress. We found that Cx43 HCs mediate the exchange of oxidants and antioxidants in lens epithelial cells undergoing oxidative stress. These transporting activities facilitate a reduction in intracellular ROS accumulation and maintain intracellular glutathione levels through the exchange of redox metabolites and changes in anti-oxidative gene expression. Additionally, Cx43 HCs can be regulated by the intracellular redox state, and this regulation is mediated by residue Cys260 located at the Cx43 C-terminus [19]. All these findings demonstrate that GJs between lens fiber cells are an important mechanism for maintaining lens transparency by transmitting redox metabolites and that HCs assist in delivering nutrients and anti-oxidants into lens epithelial and fiber cells. 

Posterior capsular opacification (PCO), a common complication following cataract surgery, results from the proliferation and degenerative fibrosis of residual lens epithelial cells [44]. The type-2 EMT and fibrosis are closely related to lens epithelial cells. Numerous studies have implicated transforming growth factor-β (TGF-β) in the fibrotic formation of PCO, including isoforms of TGFβ1 and TGFβ2 [45,46]. Additionally, there is a link between the inflammatory response to a lens injury and the mechanisms responsible for TGFβ1/β2 activation and the induction of fibrosis [47,48]. Moreover, cell adhesion signaling has also been implicated in the regulation of the EMT and fibrosis in the lens [49]. However, there is limited knowledge regarding the role of connexin channels in fibrosis. It is not clear whether GJs/HCs participate in the TGF-β-induced EMT of lens epithelial cells in response to an injury, including capsular scarring after cataract lens removal and intraocular lens implantation. Although TGF-β1 at a low concentration of 0.4 ng/mL markedly increases the expression of EMT markers, this amount is not sufficient to enhance GJ intercellular communication (GJIC) in chick embryonic lens epithelial cells. This finding has led to the deduction that the upregulation of GJIC by TGF-β is not an obvious downstream consequence of the TGF-β-induced EMT [50]. 

#### 2.1.3. Retina

Connexins are present in the five neuronal types of the retina, vascular endothelial cells, pericytes, glial cells, and the retinal pigment epithelium (RPE) [51,52]. The neuronal types of the retina are composed of photoreceptors and horizontal, bipolar, amacrine, and ganglion cells. There is a large array of connexins on these cells in mice and humans, with previous research demonstrating the presence of several Cx subtypes, including Cx35, Cx36, Cx43, Cx45, Cx57, Cx59, among others [52,53,54,55]. Furthermore, studies have largely focused on the role connexins play in material transport and signaling transmission. Cxs are reported to play crucial roles in the fibrotic development of several chronic retinal diseases, such as diabetic retinopathy (DR), retinal ischemia, age-related macular degeneration (AMD), and RPE-related diseases [51,56]. It is also worth noting that all these diseases are associated with astrocytes, Müller cells, microglia, RPE, and vessel endothelial cells in conjunction with inflammatory processes and vascular responses [51]. However, the participation of neuronal retinal Cxs in wound healing and fibrosis has not been directly reported.

Cx43, the most ubiquitously expressed isoform in these three types of glial cells, RPE, and endothelial cells, plays an essential role in multiple cellular processes [53,57,58]. As an example, Tien et al. previously reported findings indicating that the high glucose-induced downregulation of Cx43 expression and GJIC may contribute to the breakdown of endothelial barrier tight junctions associated with DR [59]. They then showed that Cx43 protein expression and GJIC were significantly reduced in diabetic retinas compared to non-diabetic retinas. These results signify that the reduction in Cx43 is associated with increased vascular cell death in human diabetic retinas [60]. In other studies, Toychiev et al. investigated the outcome of an increase in Cx43 expression and GJ coupling during an acute ischemia/reperfusion injury, which exacerbated ganglion cell loss. The inhibition of astrocytic Cx43 channels may represent a useful strategy for promoting RGC survival in pathologic conditions [61]. 

GJs and functional HCs are major focus areas of prior research. Cx HCs have a low probability of being open under normal physiological conditions. However, when open in an undocked form, these HCs allow for the ubiquitous release of paracrine and autocrine signals [62,63]. Overactivated HCs have been associated with secondary tissue injuries, including edema, compromised vascular integrity, and retinal damage, by allowing the passage of molecules and ions between the cell cytoplasm and the extracellular milieu. They can also affect downstream GJ communication [51,56,64]. During inflammation, HC-mediated ATP release is highly prominent [65]. Certain peptides, such as low concentrations of Peptide5 and Gap19, have been used to block Cx43 HCs specifically without affecting GJ coupling. These tools appear to provide viable therapeutic options for treating retinal ischemia and retinal inflammatory diseases associated with Cx43 HC opening [62,66,67]. Meanwhile, the novel benzopyran derivative Tonabersat, a sodium channel blocker that also inhibits Cx43 HCs, is used in some inflammatory-related retinal diseases, including AMD. Previous work has shown that orally administered Tonabersat improved clinical signs in animal models of both DR and dry AMD and was associated with reduced inflammation. It was discovered that Tonabersat significantly preserved the function of the retina, particularly the function of photoreceptors and bipolar cells in the inner retina [68]. Tonabersat has also been shown to prevent the formation of NOD-like receptor protein 3 (NLRP3), an ATP-mediated inflammasome, and the cleaved caspase-1 complex assembly, as well as the release of the proinflammatory cytokines IL-1β, VEGF, and IL-6 [69]. Non-obese diabetic mice have been used to evaluate the ocular safety and efficacy of Tonabersat [70]. Overall, drug treatment significantly reduces macrovascular abnormalities, hyperreflective foci, sub-retinal fluid accumulation, vascular leakage, inflammation, and inflammasome activation. Lyon H et al. have found that Tonabersat also attenuates both TGF-β2 release and RPE EMTs under disease-mimicking conditions, providing a therapeutic target for diseases, such as DR, underlined by the EMT [71]. The demonstrated efficacy of Tonabersat could be attributed in part to the inhibition of Cx43 HCs. All these findings suggest that Cx HCs inhibitors may be effective tools and hold potential as therapeutics for multiple eye diseases related to inflammation or fibrosis.

### 2.2. Connexin GJ/HCs in the Central Nervous System

A disruption of the balance of GJs, HCs, and Cxs affects tissue homeostasis and allows cells to progress toward pathological conditions with varying degrees of severity in the central nervous system (CNS) [72]. The most abundant Cx in the brain is Cx43. Cx43 is prominently expressed in astrocytes but is also present in microglial cells [73,74]. Neuronal survival and physiological functions strictly depend on the maintenance of the blood–brain barrier (BBB). Astrocytes, which establish an elaborate network at the BBB, play critical roles in neuroprotection [75,76]. 

**Figure 1:**
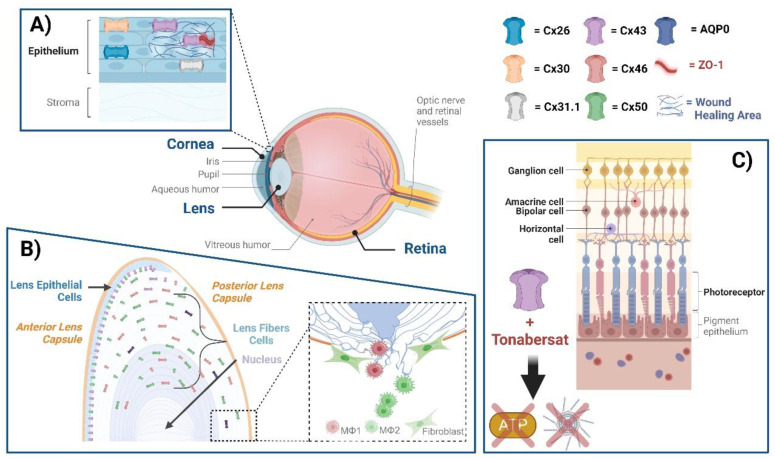
Connexin GJs and HCs in eye diseases. The eye comprises the cornea, lens, retina, and several other subcomponents, within which various connexins exist. (**A**) Cx26, Cx30, Cx31.1, and Cx43 mediate GJ communication in the human corneal epithelium. ZO-1 is a scaffolding protein that plays a role in regulating GJ assembly. A disruption of Cx43 interaction with ZO-1 leads to the enhancement of GJs; targeting this disruption could be a means of enhancing wound healing. (**B**) Cx50 and AQP0, located at lens fibers, respectively, mediate cell–cell adhesion, maintaining lens fiber integrity. Deficiency of Cx50 and AQP0 leads to a loss in cell–cell adhesion, resulting in alterations of lens structures. At the posterior part of the lens, newly formed fibers cannot bundle properly, leading to lens posterior extrusion and capsule rupture. Macrophages are recruited to the vitreous cavity adjacent to the ruptured posterior capsule. M1 macrophages mainly mediate the removal of the tissue mass, while M2 macrophages play a crucial role in posterior capsule sealing and fibrosis. (**C**) The neuronal types of the retina are composed of photoreceptors, horizontal cells, bipolar, amacrine, and ganglion cells, all of which contain connexins. The participation of neuronal retinal Cxs in wound healing and fibrosis has not been directly reported. Tonabersat, a sodium channel blocker inhibiting Cx43 HCs, is used in some inflammatory-related retinal diseases. Tonabersat has also been shown to prevent the formation of NOD-like receptor protein 3 (NLRP3), an ATP-mediated inflammasome, as well as the release of other proinflammatory cytokines. Throughout the lens tissue, targeting Cxs for inflammation or fibrosis may hold therapeutic potential.

Cx HCs form an integrated network of channels in neurons and astrocytes. Most cell types in the CNS exhibit HC activity, while most studies revolve around Cx43 HCs in astrocytes [76]. The Cx43-HC-mediated release of gliotransmitters (ATP/glutamate) results in structural neuronal alterations and increased oxidative stress [77]. While strategies that promote axonal growth are still limited, other treatment paradigms that limit secondary damage, especially maintaining a balance between pro- and anti-inflammatory factors in incomplete spinal cord injuries (SCIs), can potentially allow patients to retain greater function post-injury [78,79]. During inflammation, Ca^2+^ signaling in the astrocyte network is elevated, resulting in increased ATP production and release through the opening of HCs. ATP stimulates purinoceptors, leading to increased Ca^2+^ release from internal stores [80]. This extracellular Ca^2+^ signaling attenuates intercellular Ca^2+^ signaling, causing reduced cell communication via GJs [81,82]. Similar to the treatment of retinal diseases mentioned before, GJ and HC inhibitors strongly stimulate research toward the development of new modulators to be used against CNS disorders [83]. On one hand, Cx43 blockers can be used to deal with neuropathic pain after SCIs [84,85]. On the other hand, channel inhibitors can reduce the spread of damage and improve functional recovery after an injury [86,87]. A spinal injection of Gap26 and Gap27 effectively reduces neuropathic pain symptoms (mechanical allodynia) by decreasing the release of the chemokines CCL2 and CXCL1 in the late phase (21 days) [85]. Peptide 5, by preventing HCs from opening, significantly reduces the degree of swelling and the loss of neurons in a concentration- and time-dependent manner [86]. Cx43 AsODN treatment can reduce glial cell activation, neutrophil recruitment, and the functional consequences of a spinal cord injury. Within 24 h of a compression injury, rats treated with Cx43 AsODNs scored higher than sense- and vehicle-treated controls on behavioral tests of locomotion. In rats tested for locomotor ability over the next 28 days, the initial improvement in scores was sustained [87]. Our group has developed a novel monoclonal antibody, MHC1, that targets Cx43 and specifically inhibits HC opening but has no effect on GJ coupling in two incomplete SCI mouse models. We have shown that a single antibody administration within 30 min after an SCI significantly decreased secondary injury, improved locomotion function, attenuated gliosis, preserved white and gray matter, and protected neurons [88] (Figure 1). 

### 2.3. Connexin GJ/HCs in Bone, Skin, and Other Tissues

In bone tissue, GJs and HCs help transfer signals between adjacent cells and between cells and the extracellular environment, respectively [89,90]. In contrast to reduced GJIC, there is an overactivation of Cx HCs under oxidative stress conditions [91]. As a crucial modulator of skeletal homeostasis, Cx43 contributes to bone cell survival, proliferation, and differentiation [92,93,94]. Work by Niger et al. supports the concept that Cx43 GJs permit a more homogenous and robust response of cells to fibroblast growth factor 2 (FGF2) treatment through the propagation of signals among cells. Cx43 GJs increase intercellular communication among cells downstream of FGF receptor activation, leading to an enhanced number of cells responding to FGF2 [95]. Our group has previously reported the generation of two transgenic mouse models with Cx43-dominant negative mutants: R76W (GJs are blocked, while HCs are promoted) and ∆130-136 (both GJs and HCs are blocked), to dissect the functional contribution of Cx43 GJs and HCs in osteocytes [96]. Through these models, an impairment of Cx43-formed GJs in osteocytes appears to delay osteoclastogenesis during fracture repair, while the promotion of HCs increases new callus bone formation by accelerating the healing process [97]. 

In the skin, Cx43 is expressed in both the epidermal and dermal cutaneous layers [98,99,100]. In the first 24 h post-injury, Cx43 expression decreases within the keratinocytes of the leading edge and then increases in the hyperproliferating epidermis at day 7 [98,100,101]. A loss of Cx43 GJs occurs during the epidermal wound healing process [98]. At later stages of tissue repair, during tissue remodeling (after 7 days), the upregulation of Cx43 correlates with an increase in granulation tissue maturation [102]. The roles of GJs and HCs have been extensively studied in congenital skin disorders and wound healing, and several potential Cx-based therapeutics are under development [103,104]. Connexin mimetic peptides have been demonstrated to aid wound closure by increasing cell migration rates in human skin cells [105]. The benefits of Cx43 AsODN application include reducing scarring after a cutaneous thermal injury, enhancing the reepithelialization of wounds, and increasing granulation tissue formation [106,107,108]. A recent study by Li et al. suggests that Cx43 GJ-dependent En1-lineage negative fibroblast communication plays a vital role in matrix movement and scar formation and is necessary for the patch repair of voluminous wounds [109]. Fibroblasts and myofibroblasts have dual effects on the wound healing process after an injury. Even a subtle amount of force generation and matrix deposition is beneficial for wound healing, whereas excessive force and matrix production result in tissue scarring and even a malfunction of the repaired tissues, which may lead to organ fibrosis [14,110]. Cx43 GJs and HCs play important roles in maintaining this balance. The αCT1 peptide provides benefits in the healing of skin wounds as well [33,111]. αCT1, also used in corneal disease, has advanced to pivotal phase III clinical testing for the reduction of scar formation in surgical wounds [103,112]. This synthetic peptide modulates the wound healing response by attenuating neutrophil infiltration, increasing the vascularity of the capsule tissue, reducing type I collagen deposition, and maintaining the presence of contractile myofibroblasts [113]. In a recent study, αCT1 prompted a decrease in directionality in fibroblast movement and the generation of a 3D collagen matrix post-wounding, similar to the changes observed in unwounded skin, and correlated with a long-term improvement in scar appearance [114]. 

Although the exact mechanisms for the regulatory action of Cx43 in the fibrotic response of the kidney, heart, and lung are not completely understood, research shows that Cx43 activity is related to changes in fibroblast function. However, current studies cannot determine whether these effects are dependent on GJs and/or HCs [115,116,117]. Xu et al. used GAP 26 (a Cx43 HCs inhibitor) to alleviate unilateral-ureteral-obstruction-induced fibrosis. Cx43 mediates the release of ATP from tubular epithelial cells during a renal injury, inducing peritubular macrophage pyroptosis, which subsequently leads to the release of CXCL10 and the activation of intrarenal fibroblasts, accelerating renal fibrosis [115]. In vivo studies have indicated that the ATP receptor P2x4 expressed by fibroblasts is necessary for lung fibrosis. Macrophages were found to secrete ATP in a Cx43-dependent manner after an injury, and the fibroblast cytosolic calcium response was decreased in a co-culture with Cx43 knockout macrophages. This reveals a Cx43-dependent profibrotic effect in lung macrophages and supports the further development of the fibroblast P2x4 receptor as a therapeutic target for lung fibrosis [117].

## 3. Channel-Independent Functions of Cxs in Fibrosis, EMTs, and Wound Healing

Since 1995, studies have indicated that connexins may have separate, non-channel-related functions [118]. The molecular mechanism of this novel, significant type of action by Cxs in relation to cell growth and proliferation, differentiation, injury, and healing has been investigated and is continuously being further elucidated.

### 3.1. Cell Adhesion

The Type-2 EMT is mediated by inflammatory cells and fibroblasts, leading to reduced intercellular adhesion and increased motility [14]. Cx43 is suggested to function analogously to cell adhesion molecules in mediating cellular recognition, selective neurite adhesion, and repulsion [119]. Our group previously found that the exogenous expression of Cx50 in fibroblast cells increases their adhesive properties. In contrast, the other two Cxs expressed in the lens, Cx43 and Cx46, fail to exhibit any increase in adhesion [120]. Cx50 is capable of mediating both homotypic interactions and heterotypic protein–protein or protein–membrane interactions as a cell adhesion molecule, all independent of its role in forming Cx channels [120,121]. Moreover, the second extracellular (E2) domain is identified as a critical domain for cell adhesion function, and the expression of Cx50 enhances the expression of the adhesive molecules N-cadherin and β-catenin [122]. Aquaporin 0 (AQP0), a water-channel-forming protein, is also reported to act as an adhesive molecule and is proposed to serve as a major structural protein to facilitate the formation of the ordered cellular structure in mature lens fibers [123]. We have generated an AQP0/Cx50 double knockout (dKO) mouse model to explore the cooperative roles of these two proteins in the lens in vivo [124]. It is observed that Cx50 and AQP0 proteins in lens fibers possibly form an interlocking system due to the expression of Cx50 and AQP at broader and narrower sides of lens fiber cells, respectively. The lack of these proteins leads to a smaller lens size, posterior subcapsular/polar cataracts, and an abnormal distribution of nuclei in the posterior region. Lens fibers are also extruded from the posterior lens capsule ruptured in dKO lenses. [124].

**Figure 2:**
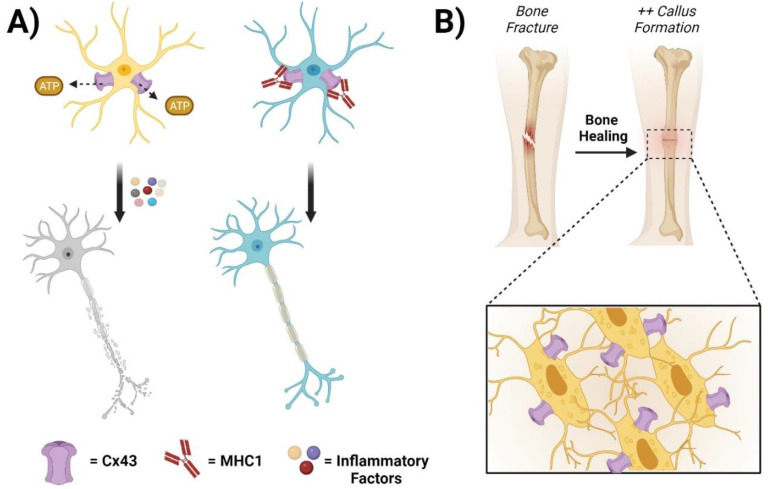
Connexin GJs and HCs in the central nervous system and bone. (**A**) Cx43 hemichannels mediate the release of ATP from edematous astrocytes, leading to the recruitment of inflammatory factors and neuronal cell death (left panels). The administration of MHC1, a monoclonal antibody that specifically inhibits Cx43 HCs but has no effect on GJs, significantly improves neuronal recovery after a traumatic spinal cord injury (right panels). (**B**) Cx43 HCs contribute to bone cell survival, proliferation, and differentiation. During fracture repair, promoting Cx43 HCs in osteocytes increases callus bone formation and accelerates the healing process.

**Figure 3:**
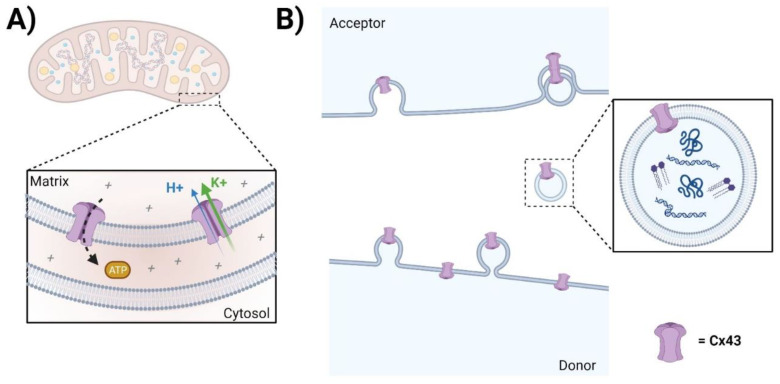
Channel-independent functions of Cx. (**A**) The role of mtCx43 is not fully understood; however, some studies suggest that mtCx43 may influence cellular processes related to tissue injury, inflammation, and fibrosis. Notably, ATP production and ROS generation are known to be involved in tissue injury and inflammation. (**B**) Annular Cx43 HC-containing vesicles can form Cx43 GJs with acceptor cells, serving as a mechanism for facilitating internalization. However, whether and how Cx43-containing exosomes contribute to injury repair, inflammation, and fibrosis remains largely elusive.

Moreover, we have intriguingly observed that in dKO lenses, the adhesion-losing lens fiber cells are extruded from the posterior capsular and form a “tail-like” tissue containing delayed regressed hyaloid vessels, fibrotic tissue, and macrophages [125]. M1 macrophages delivered by hyaloid vessels appear to mediate the clearance of the “tail-like” fiber tissue mass outside the lens at the initial stage. In the later stage, the necrotic fiber cells promote the polarization and transition to M2 macrophages inside the lens, leading to increased EMTs (Figure 2), fibrosis, and posterior capsule rupture sealing (Figure 3) [125]. During the healing process, the expression of E-cadherin decreases, whereas N-cadherin increases in the lens equatorial region of dKO lenses [125]. This suggests that macrophage-associated EMTs and fibrosis are directly involved in posterior capsule sealing and the thickening of lens epithelial cells. M2 macrophages have been reported to be closely associated with fibrosis and EMTs [126]. Intravitreal injections of an M2 macrophage activator, colony-stimulating factor 1 (CSF-1), or a CSF-1 receptor inhibitor, respectively, accelerate or delay the sealing process and fibrosis [125]. This observation provides direct evidence highlighting the critical role played by macrophages in response to damaged lens cells in an immune-privileged organ.

As a relatively new area of study, recent research has emerged challenging the definition of the lens as an immune-privileged organ. It has been shown that there is surveillance of the lens by immune cells in an adaptive response to lens degeneration and a repair response by immune cells that are activated upon lens wounding [127,128,129]. In these studies, reported by Dr. Sue Menko’s lab, the resident immune cells of the lens, located interspersed between the lens epithelium, and surveillance immune cells, which travel along the ciliary zonules from the ciliary body, are identified as the source of immune cells in association with the lens. Additionally, hyalocytes, located in the vitreous humor, are also a potential source of the immune cells that surveil the adult lens [48,130]. Macrophages exhibiting pro-wound healing, pro-fibrotic, anti-fibrotic, pro-resolving, and tissue-regenerating characteristics are commonly mentioned in the literature [131]. One of the challenges is how to regulate pro-inflammatory and anti-inflammatory macrophages at key time points to reduce injury and accelerate healing.

### 3.2. Mitochondrial Cx43

Mitochondrial Cx43 (mtCx43) was first discovered in endothelial cells [132]. Subsequently, other groups have confirmed the presence of Cx43 within mitochondria of various origins, including brown adipose tissue, astrocytes, and liver [133,134,135]. However, most research has been focused on heart tissue [136,137,138]. As of now, there are still no patch-clamp data available on Cx43-formed channels in mitoplasts [139]. Instead, putative Cx43-formed channel-like structures within mitochondria have been investigated using indirect methods [140,141]. The role of mtCx43 is not fully understood; however, some studies suggest that mtCx43 may influence cellular processes related to tissue injury, inflammation, and fibrosis. [142,143]. For example, the mechanism of the fibroblast growth factor 2-triggered subsarcolemmal protection of the heart from ischemia- and reperfusion-induced cell death requires mtCx43 channel function. It is associated with increased mtCx43 levels, as well as mtCx43 phosphorylation at the protein kinase Cε target sites S262 and S368 [144]. Studies largely support the role of mtCx43 as a critical modulator of apoptosis. Recent work from our group demonstrates the translocation of Cx43 to mitochondria in bone osteocytes after oxidative damage by promoting ATP production through increased mitochondrial membrane potential and enhancement of the proton gradient through its interaction with mitochondrial complex V [141]. Additionally, the increased ATP level in the cytosol protects the cells against oxidative stress, which depletes cytosolic ATP, leading to cell apoptosis [141]. ATP production and ROS generation are known to be involved in tissue injury and inflammation [145,146]. The data on the exact contribution of mtCx43 are scarce; more work is needed to determine other possible roles of mtCx43 in the future.

### 3.3. Exosomes

Small extracellular vesicles (sEVs), including microvesicles and exosomes, are membrane-based structures that carry and deliver bioactive molecules [147]. Osteoarthritis (OA) is a heterogeneous disease with several molecular and clinical phenotypes [148]. In OA, the downregulation of Cx43 in osteoarthritic chondrocytes restores chondrocyte redifferentiation and reduces the propensity of chondrocytes to undergo cellular senescence by downregulating p53/p21 and inhibiting the nuclear translocation of nuclear factor-kappa B(NF-κB) [149,150]. Research shows that sEVs released by human OA-derived chondrocytes contain high levels of Cx43 and induce a senescent phenotype in targeted chondrocytes, synovial cells, and bone cells, contributing to the formation of an inflammatory and degenerative joint environment through the secretion of senescence-associated secretory-associated phenotype (SASP) molecules [151]. Another study shows that exosomes derived from glioma cells under hypoxia promote the proliferation, tube formation, and migration of human umbilical vein endothelial cells through upregulated exosomal Cx43 [152]. A study by Villamizar et al. found that exosomes derived from mesenchymal stem cells may offer a hopeful therapy for patients afflicted with cystic fibrosis (CF). CF is an autosomal recessive genetic disease caused by mutations in the cystic fibrosis transmembrane conductance regulator (CFTR) gene. It is worth noting that Cx43 appears to be required for the functional release of the CFTR promoter from exosomes [153]. However, whether and how Cx43-containing exosomes contribute to injury repair, inflammation, and fibrosis remains largely elusive.

### 3.4. Other Channel-Independent Functions of Cxs

In recent years, non-channel functions have emerged for Cx43, many of which are linked to cytoskeletal dynamics and the ECM, showing that Cx43 plays diverse roles in coordinating wound closure events [154]. Using a 3D culture model, researchers have shown that channel-independent functions of Cx43 distinctly regulate wound healing-related gene expression patterns in skin and gingival fibroblasts [155]. Cx43 is phosphorylated at multiple serine residues in various cell types [156]. The evidence indicates that the protein kinase C (PKC)-dependent phosphorylation of Cx43 at S368 creates dynamic communication compartments that can temporally and spatially regulate wound healing [157]. Srisakuldee et al. revealed that Cx43 phosphorylated at S262 and S368 represents a state of increased resistance to cardiac ischemic injury, and this process is shown to be dependent on PKC activity [158,159]. The exact mechanisms for the regulatory action of Cx43 in the fibrotic response are not entirely understood, so we cannot proclaim that these effects are dependent on GJs/HCs or independent channels, and they remain areas for future studies.

## 4. Concluding Remarks

Since the first reports of Cxs, recognized initially as junction proteins, in the 1970s [160,161], they have remained a highly active research field in recent decades, particularly in the area of wound healing and injury repair. Though, at the core, functioning as a means of connecting two cells, the purpose of Cx proteins expands to paracrine signaling through their GJ and HC activities, adhesion, and their role in the regulation of mitochondrial dynamics. Interestingly, much mechanistic insight into the role of Cx GJs and HCs following an injury and during the repair process has also been obtained. Although the precise functions and regulatory mechanisms of Cxs’ channel-dependent and independent roles in fibrosis, the EMT, and wound healing remain largely obscure and need further investigation, they have still been heavily regarded as valuable therapeutic targets for this range of diseases. Future studies aim to build on these findings for eventual implementation of highly efficacious therapies targeting Cxs and Cx channels, mitigating organ dysfunction and conditions resulting from fibrosis.

## References

[1] Beyer E.C., Berthoud V.M. (2018). Gap junction gene and protein families: Connexins, innexins, and pannexins. Biochim. Biophys. Acta Biomembr..

[2] Laird D.W., Lampe P.D. (2018). Therapeutic strategies targeting connexins. Nat. Rev. Drug Discov..

[3] Beyer E.C., Paul D.L., Goodenough D.A. (1987). Connexin43: A protein from rat heart homologous to a gap junction protein from liver. J. Cell Biol..

[4] Kumar N.M., Gilula N.B. (1996). The gap junction communication channel. Cell.

[5] Yeager M., Harris A.L. (2007). Gap junction channel structure in the early 21st century: Facts and fantasies. Curr. Opin. Cell Biol..

[6] Saez J.C., Berthoud V.M., Branes M.C., Martinez A.D., Beyer E.C. (2003). Plasma membrane channels formed by connexins: Their regulation and functions. Physiol. Rev..

[7] Goodenough D.A. (1975). The structure of cell membranes involved in intercellular communication. Am. J. Clin. Pathol..

[8] Delmar M., Laird D.W., Naus C.C., Nielsen M.S., Verselis V.K., White T.W. (2018). Connexins and Disease. Cold Spring Harb. Perspect. Biol..

[9] Goodenough D.A., Goliger J.A., Paul D.L. (1996). Connexins, connexons, and intercellular communication. Annu. Rev. Biochem..

[10] Weiskirchen R., Weiskirchen S., Tacke F. (2019). Organ and tissue fibrosis: Molecular signals, cellular mechanisms and translational implications. Mol. Asp. Med..

[11] Thiery J.P., Acloque H., Huang R.Y., Nieto M.A. (2009). Epithelial-mesenchymal transitions in development and disease. Cell.

[12] Dongre A., Weinberg R.A. (2019). New insights into the mechanisms of epithelial-mesenchymal transition and implications for cancer. Nat. Rev. Mol. Cell Biol..

[13] Kalluri R., Weinberg R.A. (2009). The basics of epithelial-mesenchymal transition. J. Clin. Investig..

[14] Marconi G.D., Fonticoli L., Rajan T.S., Pierdomenico S.D., Trubiani O., Pizzicannella J., Diomede F. (2021). Epithelial-Mesenchymal Transition (EMT): The Type-2 EMT in Wound Healing, Tissue Regeneration and Organ Fibrosis. Cells.

[15] Chanson M., Derouette J.P., Roth I., Foglia B., Scerri I., Dudez T., Kwak B.R. (2005). Gap junctional communication in tissue inflammation and repair. Biochim. Biophys. Acta.

[16] Goodenough D.A., Paul D.L. (2003). Beyond the gap: Functions of unpaired connexon channels. Nat. Rev. Mol. Cell Biol..

[17] Cogliati B., Mennecier G., Willebrords J., Da Silva T.C., Maes M., Pereira I.V.A., Crespo-Yanguas S., Hernandez-Blazquez F.J., Dagli M.L.Z., Vinken M. (2016). Connexins, Pannexins, and Their Channels in Fibroproliferative Diseases. J. Membr. Biol..

[18] Vinken M., Decrock E., Vanhaecke T., Leybaert L., Rogiers V. (2012). Connexin43 signaling contributes to spontaneous apoptosis in cultures of primary hepatocytes. Toxicol. Sci..

[19] Quan Y., Du Y., Wu C., Gu S., Jiang J.X. (2021). Connexin hemichannels regulate redox potential via metabolite exchange and protect lens against cellular oxidative damage. Redox Biol..

[20] Leo-Macias A., Agullo-Pascual E., Delmar M. (2016). The cardiac connexome: Non-canonical functions of connexin43 and their role in cardiac arrhythmias. Semin. Cell Dev. Biol..

[21] Shurman D.L., Glazewski L., Gumpert A., Zieske J.D., Richard G. (2005). In vivo and in vitro expression of connexins in the human corneal epithelium. Investig. Ophthalmol. Vis. Sci..

[22] Williams K., Watsky M. (2002). Gap junctional communication in the human corneal endothelium and epithelium. Curr. Eye Res..

[23] Zhang J., Green C.R., Mugisho O.O. (2021). Cell transdifferentiation in ocular disease: Potential role for connexin channels. Exp. Cell Res..

[24] D’Hondt C., Iyyathurai J., Himpens B., Leybaert L., Bultynck G. (2014). Cx43-hemichannel function and regulation in physiology and pathophysiology: Insights from the bovine corneal endothelial cell system and beyond. Front. Physiol..

[25] Nakano Y., Oyamada M., Dai P., Nakagami T., Kinoshita S., Takamatsu T. (2008). Connexin43 knockdown accelerates wound healing but inhibits mesenchymal transition after corneal endothelial injury in vivo. Investig. Ophthalmol. Vis. Sci..

[26] Grupcheva C.N., Laux W.T., Rupenthal I.D., McGhee J., McGhee C.N., Green C.R. (2012). Improved corneal wound healing through modulation of gap junction communication using connexin43-specific antisense oligodeoxynucleotides. Investig. Ophthalmol. Vis. Sci..

[27] Giepmans B.N.G., Verlann I., Hengeveld T., Janssen H., Calafat J., Falk M.M., Moolenaar W.H. (2001). Gap junction protein connexin-43 interacts directly with microtubules. Curr. Biol..

[28] Hunter A.L., Choy J.C., Granville D.J. (2005). Detection of apoptosis in cardiovascular diseases. Methods Mol. Med..

[29] Rhett J.M., Jourdan J., Gourdie R.G. (2011). Connexin 43 connexon to gap junction transition is regulated by zonula occludens-1. Mol. Biol. Cell.

[30] Sorgen P.L., Duffy H.S., Sahoo P., Coombs W., Delmar M., Spray D.C. (2004). Structural changes in the carboxyl terminus of the gap junction protein connexin43 indicates signaling between binding domains for c-Src and zonula occludens-1. J. Biol. Chem..

[31] Hunter A.W., Barker R.J., Zhu C., Gourdie R.G. (2005). Zonula occludens-1 alters connexin43 gap junction size and organization by influencing channel accretion. Mol. Biol. Cell.

[32] Moore K., Bryant Z.J., Ghatnekar G., Singh U.P., Gourdie R.G., Potts J.D. (2013). A synthetic connexin 43 mimetic peptide augments corneal wound healing. Exp. Eye Res..

[33] Rhett J.M., Ghatnekar G.S., Palatinus J.A., O’Quinn M., Yost M.J., Gourdie R.G. (2008). Novel therapies for scar reduction and regenerative healing of skin wounds. Trends Biotechnol..

[34] Ormonde S., Chou C.Y., Goold L., Petsoglou C., Al-Taie R., Sherwin T., McGhee C.N., Green C.R. (2012). Regulation of connexin43 gap junction protein triggers vascular recovery and healing in human ocular persistent epithelial defect wounds. J. Membr. Biol..

[35] Evans W.H., Boitano S. (2001). Connexin mimetic peptides: Specific inhibitors of gap-junctional intercellular communication. Biochem. Soc. Trans..

[36] Elbadawy H.M., Mirabelli P., Xeroudaki M., Parekh M., Bertolin M., Breda C., Cagini C., Ponzin D., Lagali N., Ferrari S. (2016). Effect of connexin 43 inhibition by the mimetic peptide Gap27 on corneal wound healing, inflammation and neovascularization. Br. J. Pharmacol..

[37] Beyer E.C., Berthoud V.M. (2014). Connexin hemichannels in the lens. Front. Physiol..

[38] Berthoud V.M., Gao J., Minogue P.J., Jara O., Mathias R.T., Beyer E.C. (2020). Connexin Mutants Compromise the Lens Circulation and Cause Cataracts through Biomineralization. Int. J. Mol. Sci..

[39] Jiang J.X. (2010). Gap junctions or hemichannel-dependent and independent roles of connexins in cataractogenesis and lens development. Curr. Mol. Med..

[40] Ren Q., Riquelme M.A., Xu J., Yan X., Nicholson B.J., Gu S., Jiang J.X. (2013). Cataract-causing mutation of human connexin 46 impairs gap junction, but increases hemichannel function and cell death. PLoS ONE.

[41] Shi W., Riquelme M.A., Gu S., Jiang J.X. (2018). Connexin hemichannels mediate glutathione transport and protect lens fiber cells from oxidative stress. J. Cell Sci..

[42] Liu J., Riquelme M.A., Li Z., Li Y., Tong Y., Quan Y., Pei C., Gu S., Jiang J.X. (2020). Mechanosensitive collaboration between integrins and connexins allows nutrient and antioxidant transport into the lens. J. Cell Biol..

[43] Du Y., Tong Y., Quan Y., Wang G., Cheng H., Gu S., Jiang J.X. (2023). Protein kinase A activation alleviates cataract formation via increased gap junction intercellular communication. iScience.

[44] Apple D.J., Solomon K.D., Tetz M.R., Assia E.I., Holland E.Y., Legler U.F., Tsai J.C., Castaneda V.E., Hoggatt J.P., Kostick A.M. (1992). Posterior capsule opacification. Surv. Ophthalmol..

[45] Wormstone I.M., Tamiya S., Anderson I., Duncan G. (2002). TGF-beta2-induced matrix modification and cell transdifferentiation in the human lens capsular bag. Investig. Ophthalmol. Vis. Sci..

[46] Boswell B.A., Korol A., West-Mays J.A., Musil L.S. (2017). Dual function of TGFbeta in lens epithelial cell fate: Implications for secondary cataract. Mol. Biol. Cell.

[47] Jiang J., Shihan M.H., Wang Y., Duncan M.K. (2018). Lens Epithelial Cells Initiate an Inflammatory Response Following Cataract Surgery. Investig. Ophthalmol. Vis. Sci..

[48] Stepp M.A., Menko A.S. (2021). Immune responses to injury and their links to eye disease. Transl. Res..

[49] Taiyab A., West-Mays J. (2022). Lens Fibrosis: Understanding the Dynamics of Cell Adhesion Signaling in Lens Epithelial-Mesenchymal Transition. Front. Cell Dev. Biol..

[50] Boswell B.A., VanSlyke J.K., Musil L.S. (2010). Regulation of lens gap junctions by Transforming Growth Factor beta. Mol. Biol. Cell.

[51] Danesh-Meyer H.V., Zhang J., Acosta M.L., Rupenthal I.D., Green C.R. (2016). Connexin43 in retinal injury and disease. Prog. Retin. Eye Res..

[52] Gonzalez-Casanova J., Schmachtenberg O., Martinez A.D., Sanchez H.A., Harcha P.A., Rojas-Gomez D. (2021). An Update on Connexin Gap Junction and Hemichannels in Diabetic Retinopathy. Int. J. Mol. Sci..

[53] Sohl G., Joussen A., Kociok N., Willecke K. (2010). Expression of connexin genes in the human retina. BMC Ophthalmol..

[54] Harrison K.R., Chervenak A.P., Resnick S.M., Reifler A.N., Wong K.Y. (2021). Amacrine Cells Forming Gap Junctions With Intrinsically Photosensitive Retinal Ganglion Cells: ipRGC Types, Neuromodulator Contents, and Connexin Isoform. Investig. Ophthalmol. Vis. Sci..

[55] Guldenagel M., Sohl G., Plum A., Traub O., Teubner B., Weiler R., Willecke K. (2000). Expression patterns of connexin genes in mouse retina. J. Comp. Neurol..

[56] Roy S., Jiang J.X., Li A.F., Kim D. (2017). Connexin channel and its role in diabetic retinopathy. Prog. Retin. Eye Res..

[57] Kerr N.M., Johnson C.S., Green C.R., Danesh-Meyer H.V. (2011). Gap junction protein connexin43 (GJA1) in the human glaucomatous optic nerve head and retina. J. Clin. Neurosci..

[58] Mendes-Jorge L., Llombart C., Ramos D., Lopez-Luppo M., Valenca A., Nacher V., Navarro M., Carretero A., Mendez-Ferrer S., Rodriguez-Baeza A. (2012). Intercapillary bridging cells: Immunocytochemical characteristics of cells that connect blood vessels in the retina. Exp. Eye Res..

[59] Tien T., Barrette K.F., Chronopoulos A., Roy S. (2013). Effects of high glucose-induced Cx43 downregulation on occludin and ZO-1 expression and tight junction barrier function in retinal endothelial cells. Investig. Ophthalmol. Vis. Sci..

[60] Tien T., Muto T., Zhang J., Sohn E.H., Mullins R.F., Roy S. (2016). Association of reduced Connexin 43 expression with retinal vascular lesions in human diabetic retinopathy. Exp. Eye Res..

[61] Toychiev A.H., Batsuuri K., Srinivas M. (2021). Gap Junctional Coupling Between Retinal Astrocytes Exacerbates Neuronal Damage in Ischemia-Reperfusion Injury. Investig. Ophthalmol. Vis. Sci..

[62] Abudara V., Bechberger J., Freitas-Andrade M., De Bock M., Wang N., Bultynck G., Naus C.C., Leybaert L., Giaume C. (2014). The connexin43 mimetic peptide Gap19 inhibits hemichannels without altering gap junctional communication in astrocytes. Front. Cell Neurosci..

[63] Orellana J.A., Retamal M.A., Moraga-Amaro R., Stehberg J. (2016). Role of Astroglial Hemichannels and Pannexons in Memory and Neurodegenerative Diseases. Front. Integr. Neurosci..

[64] Acosta M.L., Mat Nor M.N., Guo C.X., Mugisho O.O., Coutinho F.P., Rupenthal I.D., Green C.R. (2021). Connexin therapeutics: Blocking connexin hemichannel pores is distinct from blocking pannexin channels or gap junctions. Neural Regen. Res..

[65] Bennett M.V., Garre J.M., Orellana J.A., Bukauskas F.F., Nedergaard M., Saez J.C. (2012). Connexin and pannexin hemichannels in inflammatory responses of glia and neurons. Brain Res..

[66] Guo C.X., Mat Nor M.N., Danesh-Meyer H.V., Vessey K.A., Fletcher E.L., O’Carroll S.J., Acosta M.L., Green C.R. (2016). Connexin43 Mimetic Peptide Improves Retinal Function and Reduces Inflammation in a Light-Damaged Albino Rat Model. Investig. Ophthalmol. Vis. Sci..

[67] Danesh-Meyer H.V., Kerr N.M., Zhang J., Eady E.K., O’Carroll S.J., Nicholson L.F., Johnson C.S., Green C.R. (2012). Connexin43 mimetic peptide reduces vascular leak and retinal ganglion cell death following retinal ischaemia. Brain.

[68] Mat Nor M.N., Rupenthal I.D., Green C.R., Acosta M.L. (2020). Connexin Hemichannel Block Using Orally Delivered Tonabersat Improves Outcomes in Animal Models of Retinal Disease. Neurotherapeutics.

[69] Lyon H., Shome A., Rupenthal I.D., Green C.R., Mugisho O.O. (2020). Tonabersat Inhibits Connexin43 Hemichannel Opening and Inflammasome Activation in an In Vitro Retinal Epithelial Cell Model of Diabetic Retinopathy. Int. J. Mol. Sci..

[70] Mugisho O.O., Aryal J., Shorne A., Lyon H., Acosta M.L., Green C.R., Rupenthal I.D. (2023). Orally Delivered Connexin43 Hemichannel Blocker, Tonabersat, Inhibits Vascular Breakdown and Inflammasome Activation in a Mouse Model of Diabetic Retinopathy. Int. J. Mol. Sci..

[71] Lyon H., Yin N., Rupenthal I.D., Green C.R., Mugisho O.O. (2022). Blocking connexin43 hemichannels prevents TGF-beta2 upregulation and epithelial-mesenchymal transition in retinal pigment epithelial cells. Cell Biol. Int..

[72] Vicario N., Zappala A., Calabrese G., Gulino R., Parenti C., Gulisano M., Parenti R. (2017). Connexins in the Central Nervous System: Physiological Traits and Neuroprotective Targets. Front. Physiol..

[73] Giaume C., Theis M. (2010). Pharmacological and genetic approaches to study connexin-mediated channels in glial cells of the central nervous system. Brain Res. Rev..

[74] Panattoni G., Amoriello R., Memo C., Thalhammer A., Ballerini C., Ballerini L. (2021). Diverse inflammatory threats modulate astrocytes Ca(2+) signaling via connexin43 hemichannels in organotypic spinal slices. Mol. Brain.

[75] Sanmarco L.M., Polonio C.M., Wheeler M.A., Quintana F.J. (2021). Functional immune cell-astrocyte interactions. J. Exp. Med..

[76] Vicario N., Parenti R. (2022). Connexins Signatures of the Neurovascular Unit and Their Physio-Pathological Functions. Int. J. Mol. Sci..

[77] Retamal M.A., Froger N., Palacios-Prado N., Ezan P., Saez P.J., Saez J.C., Giaume C. (2007). Cx43 hemichannels and gap junction channels in astrocytes are regulated oppositely by proinflammatory cytokines released from activated microglia. J. Neurosci..

[78] Liu Y.D., Tang G., Qian F., Liu L., Huang J.R., Tang F.R. (2021). Astroglial Connexins in Neurological and Neuropsychological Disorders and Radiation Exposure. Curr. Med. Chem..

[79] Goldshmit Y., Jona G., Schmukler E., Solomon S., Pinkas-Kramarski R., Ruban A. (2018). Blood Glutamate Scavenger as a Novel Neuroprotective Treatment in Spinal Cord Injury. J. Neurotrauma.

[80] Zorec R., Araque A., Carmignoto G., Haydon P.G., Verkhratsky A., Parpura V. (2012). Astroglial excitability and gliotransmission: An appraisal of Ca2+ as a signalling route. ASN Neuro.

[81] Hansson E., Skiöldebrand E. (2015). Coupled cell networks are target cells of inflammation, which can spread between different body organs and develop into systemic chronic inflammation. J. Inflamm..

[82] Karpuk N., Burkovetskaya M., Fritz T., Angle A., Kielian T. (2011). Neuroinflammation leads to region-dependent alterations in astrocyte gap junction communication and hemichannel activity. J. Neurosci..

[83] Abou-Mrad Z., Alomari S.O., Bsat S., Moussalem C.K., Alok K., El Houshiemy M.N., Alomari A.O., Minassian G.B., Omeis I.A. (2020). Role of connexins in spinal cord injury: An update. Clin. Neurol. Neurosurg..

[84] Chen M.J., Kress B., Han X., Moll K., Peng W., Ji R.R., Nedergaard M. (2012). Astrocytic CX43 hemichannels and gap junctions play a crucial role in development of chronic neuropathic pain following spinal cord injury. Glia.

[85] Chen G., Park C.K., Xie R.G., Berta T., Nedergaard M., Ji R.R. (2014). Connexin-43 induces chemokine release from spinal cord astrocytes to maintain late-phase neuropathic pain in mice. Brain.

[86] O’Carroll S.J., Alkadhi M., Nicholson L.F., Green C.R. (2008). Connexin 43 mimetic peptides reduce swelling, astrogliosis, and neuronal cell death after spinal cord injury. Cell Commun. Adhes..

[87] Cronin M., Anderson P.N., Cook J.E., Green C.R., Becker D.L. (2008). Blocking connexin43 expression reduces inflammation and improves functional recovery after spinal cord injury. Mol. Cell Neurosci..

[88] Zhang C., Yan Z., Maknojia A., Riquelme M.A., Gu S., Booher G., Wallace D.J., Bartanusz V., Goswami A., Xiong W. (2021). Inhibition of astrocyte hemichannel improves recovery from spinal cord injury. JCI Insight.

[89] Batra N., Kar R., Jiang J.X. (2012). Gap junctions and hemichannels in signal transmission, function and development of bone. Biochim. Biophys. Acta.

[90] Plotkin L.I., Bellido T. (2013). Beyond gap junctions: Connexin43 and bone cell signaling. Bone.

[91] Hua R., Zhang J., Riquelme M.A., Jiang J.X. (2021). Connexin Gap Junctions and Hemichannels Link Oxidative Stress to Skeletal Physiology and Pathology. Curr. Osteoporos. Rep..

[92] Bivi N., Condon K.W., Allen M.R., Farlow N., Passeri G., Brun L.R., Rhee Y., Bellido T., Plotkin L.I. (2012). Cell autonomous requirement of connexin 43 for osteocyte survival: Consequences for endocortical resorption and periosteal bone formation. J. Bone Miner. Res..

[93] Watkins M., Grimston S.K., Norris J.Y., Guillotin B., Shaw A., Beniash E., Civitelli R. (2011). Osteoblast connexin43 modulates skeletal architecture by regulating both arms of bone remodeling. Mol. Biol. Cell.

[94] Hua R., Gu S., Jiang J.X. (2022). Connexin 43 Hemichannels Regulate Osteoblast to Osteocyte Differentiation. Front. Cell Dev. Biol..

[95] Niger C., Buo A.M., Hebert C., Duggan B.T., Williams M.S., Stains J.P. (2012). ERK acts in parallel to PKCdelta to mediate the connexin43-dependent potentiation of Runx2 activity by FGF2 in MC3T3 osteoblasts. Am. J. Physiol. Cell Physiol..

[96] Xu H., Gu S., Riquelme M.A., Burra S., Callaway D., Cheng H., Guda T., Schmitz J., Fajardo R.J., Werner S.L. (2015). Connexin 43 channels are essential for normal bone structure and osteocyte viability. J. Bone Miner. Res..

[97] Chen Y., Chen M., Xue T., Li G., Wang D., Shang P., Jiang J.X., Xu H. (2019). Osteocytic connexin 43 channels affect fracture healing. J. Cell Physiol..

[98] Coutinho P., Qiu C., Frank S., Tamber K., Becker D. (2003). Dynamic changes in connexin expression correlate with key events in the wound healing process. Cell Biol. Int..

[99] Qiu C., Coutinho P., Frank S., Franke S., Law L.Y., Martin P., Green C.R., Becker D.L. (2003). Targeting connexin43 expression accelerates the rate of wound repair. Curr. Biol..

[100] Goliger J.A., Paul D.L. (1995). Wounding alters epidermal connexin expression and gap junction-mediated intercellular communication. Mol. Biol. Cell.

[101] Saitoh M., Oyamada M., Oyamada Y., Kaku T., Mori M. (1997). Changes in the expression of gap junction proteins (connexins) in hamster tongue epithelium during wound healing and carcinogenesis. Carcinogenesis.

[102] Moyer K.E., Davis A., Saggers G.C., Mackay D.R., Ehrlich H.P. (2002). Wound healing: The role of gap junctional communication in rat granulation tissue maturation. Exp. Mol. Pathol..

[103] Montgomery J., Ghatnekar G.S., Grek C.L., Moyer K.E., Gourdie R.G. (2018). Connexin 43-Based Therapeutics for Dermal Wound Healing. Int. J. Mol. Sci..

[104] Lilly E., Sellitto C., Milstone L.M., White T.W. (2016). Connexin channels in congenital skin disorders. Semin. Cell Dev. Biol..

[105] Wright C.S., van Steensel M.A., Hodgins M.B., Martin P.E. (2009). Connexin mimetic peptides improve cell migration rates of human epidermal keratinocytes and dermal fibroblasts in vitro. Wound Repair. Regen..

[106] Coutinho P., Qiu C., Frank S., Wang C.M., Brown T., Green C.R., Becker D.L. (2005). Limiting burn extension by transient inhibition of Connexin43 expression at the site of injury. Br. J. Plast. Surg..

[107] Mori R., Power K.T., Wang C.M., Martin P., Becker D.L. (2006). Acute downregulation of connexin43 at wound sites leads to a reduced inflammatory response, enhanced keratinocyte proliferation and wound fibroblast migration. J. Cell Sci..

[108] Wang C.M., Lincoln J., Cook J.E., Becker D.L. (2007). Abnormal connexin expression underlies delayed wound healing in diabetic skin. Diabetes.

[109] Wan L., Jiang D., Correa-Gallegos D., Ramesh P., Zhao J., Ye H., Zhu S., Wannemacher J., Volz T., Rinkevich Y. (2021). Connexin43 gap junction drives fascia mobilization and repair of deep skin wounds. Matrix Biol..

[110] Li B., Wang J.H. (2011). Fibroblasts and myofibroblasts in wound healing: Force generation and measurement. J. Tissue Viability.

[111] Gourdie R.G., Ghatnekar G.S., O’Quinn M., Rhett M.J., Barker R.J., Zhu C., Jourdan J., Hunter A.W. (2006). The unstoppable connexin43 carboxyl-terminus: New roles in gap junction organization and wound healing. Ann. N. Y. Acad. Sci..

[112] Grek C.L., Montgomery J., Sharma M., Ravi A., Rajkumar J.S., Moyer K.E., Gourdie R.G., Ghatnekar G.S. (2017). A Multicenter Randomized Controlled Trial Evaluating a Cx43-Mimetic Peptide in Cutaneous Scarring. J. Investig. Dermatol..

[113] Soder B.L., Propst J.T., Brooks T.M., Goodwin R.L., Friedman H.I., Yost M.J., Gourdie R.G. (2009). The connexin43 carboxyl-terminal peptide ACT1 modulates the biological response to silicone implants. Plast. Reconstr. Surg..

[114] Montgomery J., Richardson W.J., Marsh S., Rhett J.M., Bustos F., Degen K., Ghatnekar G.S., Grek C.L., Jourdan L.J., Holmes J.W. (2021). The connexin 43 carboxyl terminal mimetic peptide alphaCT1 prompts differentiation of a collagen scar matrix in humans resembling unwounded skin. FASEB J..

[115] Xu H., Wang M., Li Y., Shi M., Wang Z., Cao C., Hong Y., Hu B., Zhu H., Zhao Z. (2022). Blocking connexin 43 and its promotion of ATP release from renal tubular epithelial cells ameliorates renal fibrosis. Cell Death Dis..

[116] Rodriguez-Sinovas A., Sanchez J.A., Valls-Lacalle L., Consegal M., Ferreira-Gonzalez I. (2021). Connexins in the Heart: Regulation, Function and Involvement in Cardiac Disease. Int. J. Mol. Sci..

[117] Bhattacharyya A., Torre P., Yadav P., Boostanpour K., Chen T.Y., Tsukui T., Sheppard D., Muramatsu R., Seed R.I., Nishimura S.L. (2022). Macrophage Cx43 Is Necessary for Fibroblast Cytosolic Calcium and Lung Fibrosis After Injury. Front. Immunol..

[118] Mesnil M., Krutovskikh V., Piccoli C., Elfgang C., Traub O., Willecke K., Yamasaki H. (1995). Negative growth control of HeLa cells by connexin genes: Connexin species specificity. Cancer Res..

[119] Strauss R.E., Gourdie R.G. (2020). Cx43 and the Actin Cytoskeleton: Novel Roles and Implications for Cell-Cell Junction-Based Barrier Function Regulation. Biomolecules.

[120] Hu Z., Shi W., Riquelme M.A., Shi Q., Biswas S., Lo W.K., White T.W., Gu S., Jiang J.X. (2017). Connexin 50 Functions as an Adhesive Molecule and Promotes Lens Cell Differentiation. Sci. Rep..

[121] Li Z., Quan Y., Gu S., Jiang J.X. (2022). Beyond the Channels: Adhesion Functions of Aquaporin 0 and Connexin 50 in Lens Development. Front. Cell Dev. Biol..

[122] Li Z., Quan Y., Wang G., Ma B., Gu S., Jiang J.X. (2023). The second extracellular domain of connexin 50 is important for in cell adhesion, lens differentiation, and adhesion molecule expression. J. Biol. Chem..

[123] Chepelinsky A.B. (2009). Structural function of MIP/aquaporin 0 in the eye lens; genetic defects lead to congenital inherited cataracts. Handbook of Experimental Pharmacology.

[124] Gu S., Biswas S., Rodriguez L., Li Z., Li Y., Riquelme M.A., Shi W., Wang K., White T.W., Reilly M. (2019). Connexin 50 and AQP0 are Essential in Maintaining Organization and Integrity of Lens Fibers. Investig. Ophthalmol. Vis. Sci..

[125] Li Y., Li Z., Quan Y., Cheng H., Riquelme M.A., Li X.D., Gu S., Jiang J.X. (2021). Macrophage recruitment in immune-privileged lens during capsule repair, necrotic fiber removal, and fibrosis. iScience.

[126] Yao R.R., Li J.H., Zhang R., Chen R.X., Wang Y.H. (2018). M2-polarized tumor-associated macrophages facilitated migration and epithelial-mesenchymal transition of HCC cells via the TLR4/STAT3 signaling pathway. World J. Surg. Oncol..

[127] DeDreu J., Bowen C.J., Logan C.M., Pal-Ghosh S., Parlanti P., Stepp M.A., Menko A.S. (2020). An immune response to the avascular lens following wounding of the cornea involves ciliary zonule fibrils. FASEB J..

[128] Logan C.M., Bowen C.J., Menko A.S. (2017). Induction of Immune Surveillance of the Dysmorphogenic Lens. Sci. Rep..

[129] Menko A.S., DeDreu J., Logan C.M., Paulson H., Levin A.V., Walker J.L. (2021). Resident immune cells of the avascular lens: Mediators of the injury and fibrotic response of the lens. FASEB J..

[130] Lutty G.A., McLeod D.S. (2018). Development of the hyaloid, choroidal and retinal vasculatures in the fetal human eye. Prog. Retin. Eye Res..

[131] Wynn T.A., Vannella K.M. (2016). Macrophages in Tissue Repair, Regeneration, and Fibrosis. Immunity.

[132] Li H., Brodsky S., Kumari S., Valiunas V., Brink P., Kaide J., Nasjletti A., Goligorsky M.S. (2002). Paradoxical overexpression and translocation of connexin43 in homocysteine-treated endothelial cells. Am. J. Physiol. Heart Circ. Physiol..

[133] Kim S.N., Kwon H.J., Im S.W., Son Y.H., Akindehin S., Jung Y.S., Lee S.J., Rhyu I.J., Kim I.Y., Seong J.K. (2017). Connexin 43 is required for the maintenance of mitochondrial integrity in brown adipose tissue. Sci. Rep..

[134] Kozoriz M.G., Church J., Ozog M.A., Naus C.C., Krebs C. (2010). Temporary sequestration of potassium by mitochondria in astrocytes. J. Biol. Chem..

[135] Li Z., Chen L., Chu H., Wang W., Yang L. (2022). Estrogen alleviates hepatocyte necroptosis depending on GPER in hepatic ischemia reperfusion injury. J. Physiol. Biochem..

[136] Boengler K., Dodoni G., Rodriguez-Sinovas A., Cabestrero A., Ruiz-Meana M., Gres P., Konietzka I., Lopez-Iglesias C., Garcia-Dorado D., Di Lisa F. (2005). Connexin 43 in cardiomyocyte mitochondria and its increase by ischemic preconditioning. Cardiovasc. Res..

[137] Wang M., Smith K., Yu Q., Miller C., Singh K., Sen C.K. (2019). Mitochondrial connexin 43 in sex-dependent myocardial responses and estrogen-mediated cardiac protection following acute ischemia/reperfusion injury. Basic. Res. Cardiol..

[138] Wei X., Chang A.C.H., Chang H., Xu S., Xue Y., Zhang Y., Lei M., Chang A.C.Y., Zhang Q. (2022). Hypoglycemia-Exacerbated Mitochondrial Connexin 43 Accumulation Aggravates Cardiac Dysfunction in Diabetic Cardiomyopathy. Front. Cardiovasc. Med..

[139] Boengler K., Leybaert L., Ruiz-Meana M., Schulz R. (2022). Connexin 43 in Mitochondria: What Do We Really Know About Its Function?. Front. Physiol..

[140] Gadicherla A.K., Wang N., Bulic M., Agullo-Pascual E., Lissoni A., De Smet M., Delmar M., Bultynck G., Krysko D.V., Camara A. (2017). Mitochondrial Cx43 hemichannels contribute to mitochondrial calcium entry and cell death in the heart. Basic. Res. Cardiol..

[141] Zhang J., Riquelme M.A., Hua R., Acosta F.M., Gu S., Jiang J.X. (2022). Connexin 43 hemichannels regulate mitochondrial ATP generation, mobilization, and mitochondrial homeostasis against oxidative stress. Elife.

[142] Azarashvili T., Baburina Y., Grachev D., Krestinina O., Evtodienko Y., Stricker R., Reiser G. (2011). Calcium-induced permeability transition in rat brain mitochondria is promoted by carbenoxolone through targeting connexin43. Am. J. Physiol. Cell Physiol..

[143] Kowluru R.A. (2005). Diabetic retinopathy: Mitochondrial dysfunction and retinal capillary cell death. Antioxid. Redox Signal.

[144] Srisakuldee W., Makazan Z., Nickel B.E., Zhang F., Thliveris J.A., Pasumarthi K.B., Kardami E. (2014). The FGF-2-triggered protection of cardiac subsarcolemmal mitochondria from calcium overload is mitochondrial connexin 43-dependent. Cardiovasc. Res..

[145] Fang X., Huang T., Zhu Y., Yan Q., Chi Y., Jiang J.X., Wang P., Matsue H., Kitamura M., Yao J. (2011). Connexin43 hemichannels contribute to cadmium-induced oxidative stress and cell injury. Antioxid. Redox Signal.

[146] Huang Y., Mao Z., Zhang Z., Obata F., Yang X., Zhang X., Huang Y., Mitsui T., Fan J., Takeda M. (2019). Connexin43 Contributes to Inflammasome Activation and Lipopolysaccharide-Initiated Acute Renal Injury via Modulation of Intracellular Oxidative Status. Antioxid. Redox Signal.

[147] Varela-Eirin M., Varela-Vazquez A., Rodriguez-Candela Mateos M., Vila-Sanjurjo A., Fonseca E., Mascarenas J.L., Eugenio Vazquez M., Mayan M.D. (2017). Recruitment of RNA molecules by connexin RNA-binding motifs: Implication in RNA and DNA transport through microvesicles and exosomes. Biochim. Biophys. Acta Mol. Cell Res..

[148] Martel-Pelletier J., Barr A.J., Cicuttini F.M., Conaghan P.G., Cooper C., Goldring M.B., Goldring S.R., Jones G., Teichtahl A.J., Pelletier J.P. (2016). Osteoarthritis. Nat. Rev. Dis. Primers.

[149] Varela-Eirin M., Varela-Vazquez A., Guitian-Caamano A., Paino C.L., Mato V., Largo R., Aasen T., Tabernero A., Fonseca E., Kandouz M. (2018). Targeting of chondrocyte plasticity via connexin43 modulation attenuates cellular senescence and fosters a pro-regenerative environment in osteoarthritis. Cell Death Dis..

[150] Varela-Eirin M., Carpintero-Fernandez P., Sanchez-Temprano A., Varela-Vazquez A., Paino C.L., Casado-Diaz A., Calanas-Continente A., Mato V., Fonseca E., Kandouz M. (2020). Senolytic activity of small molecular polyphenols from olive restores chondrocyte redifferentiation and promotes a pro-regenerative environment in osteoarthritis. Aging.

[151] Varela-Eirin M., Carpintero-Fernandez P., Guitian-Caamano A., Varela-Vazquez A., Garcia-Yuste A., Sanchez-Temprano A., Bravo-Lopez S.B., Yanez-Cabanas J., Fonseca E., Largo R. (2022). Extracellular vesicles enriched in connexin 43 promote a senescent phenotype in bone and synovial cells contributing to osteoarthritis progression. Cell Death Dis..

[152] Yang Z.J., Bi Q.C., Gan L.J., Zhang L.L., Wei M.J., Hong T., Liu R., Qiu C.L., Han X.J., Jiang L.P. (2022). Exosomes Derived from Glioma Cells under Hypoxia Promote Angiogenesis through Up-regulated Exosomal Connexin 43. Int. J. Med. Sci..

[153] Villamizar O., Waters S.A., Scott T., Grepo N., Jaffe A., Morris K.V. (2021). Mesenchymal Stem Cell exosome delivered Zinc Finger Protein activation of cystic fibrosis transmembrane conductance regulator. J. Extracell. Vesicles.

[154] Lorraine C., Wright C.S., Martin P.E. (2015). Connexin43 plays diverse roles in co-ordinating cell migration and wound closure events. Biochem. Soc. Trans..

[155] Tarzemany R., Jiang G., Jiang J.X., Gallant-Behm C., Wiebe C., Hart D.A., Larjava H., Hakkinen L. (2018). Connexin 43 regulates the expression of wound healing-related genes in human gingival and skin fibroblasts. Exp. Cell Res..

[156] Musil L.S., Cunningham B.A., Edelman G.M., Goodenough D.A. (1990). Differential phosphorylation of the gap junction protein connexin43 in junctional communication-competent and -deficient cell lines. J. Cell Biol..

[157] Richards T.S., Dunn C.A., Carter W.G., Usui M.L., Olerud J.E., Lampe P.D. (2004). Protein kinase C spatially and temporally regulates gap junctional communication during human wound repair via phosphorylation of connexin43 on serine368. J. Cell Biol..

[158] Srisakuldee W., Jeyaraman M.M., Nickel B.E., Tanguy S., Jiang Z.S., Kardami E. (2009). Phosphorylation of connexin-43 at serine 262 promotes a cardiac injury-resistant state. Cardiovasc. Res..

[159] Zhou J.Z., Jiang J.X. (2014). Gap junction and hemichannel-independent actions of connexins on cell and tissue functions—An update. FEBS Lett..

[160] Dewey M.M., Barr L. (1962). Intercellular Connection between Smooth Muscle Cells: The Nexus. Science.

[161] Goodenough D.A. (1974). Bulk isolation of mouse hepatocyte gap junctions. Characterization of the principal protein, connexin. J. Cell Biol..

